# Morphology and Ultrastructure of the Accessory Glands in the Female Genital Tract of the House Cricket, *Acheta domesticus*

**DOI:** 10.1673/031.012.9901

**Published:** 2012-08-17

**Authors:** Robert Sturm

**Affiliations:** Brunnleitenweg 41, A-5061 Elsbethen, Salzburg, Austria

**Keywords:** accessory structure, cuticula intima, gland epithelium, reproductive tract, secretion

## Abstract

The accessory glands in the genital tract of female *Acheta domesticus* L. (Orthoptera: Gryllidae) were investigated in detail. The glands are situated within the 7^th^ and 8^th^ abdominal segment and lead to the genital chamber lateral to the terminal papilla of the ductus receptaculi. The shape of the gland is characterized by a complex system of tubules, including numerous ramifications. The gland's size ranges from 2 to 4 mm. The epithelium is constructed according to a simple scheme and consists of a cuticular intima at the luminal side, one layer of gland cells, and a basallamina at the outermost side. The observed morphology of the accessory glands widely corresponds with that in other cricket species (e.g., *Teleogryllus commodus*). This is also true for the structure of a single gland cell, which can be subdivided into a basal part with nucleus and intracellular cisternae, as well as an apical part with all those compartments responsible for the production of the secretion. The secretion itself may be classified as lipophilic and is produced for the first time 4 to 6 days after the imaginai moult. Several endogenic functions of the secretion are discussed (lubricant for oviposition, support for introducing the tube of the spermatophore into the ductus receptaculi, etc.).

## Introduction

Based upon comprehensive morphological studies (e.g., Happ 1984; Gillott 1988; Kaulenas 1992; Sturm and Pohlhammer 2000), the accessory glands commonly represent an essential component of the reproductive apparatus of numerous insects. Concerning male insects, the secretions released from these glands generally support the transfer of the sperm mass to the female receptaculum seminis during copulation. In the case of some insect orders, including the Orthoptera, this transfer of the spermatozoa takes place by the formation of spermatophores (i.e., acellular sperm capsules consisting of an ampulla, a tube, and sometimes a so-called spermatophylax) that are appended to the female genital tract with the help of specific attachment plates (de Wilde 1964; Tuzet 1977; Sturm 2002a, 2002b). Besides the production of paraseminal structures, the male accessory glands, among others, serve in the secretion of nutritional substances for the sperm and the release of transmitters that influence the behavior and physiology of the females (Leopold 1976; Gillott 1996).

Regarding their functions, the female accessory glands are of much less significance than their male analogs, which in some insect orders (e.g., Ephemeroptera) resulted in their complete secondary reduction (Matsuda 1976). Among some insects (e.g., *Periplaneta americana*) the released secretions are used as additional building substances for the egg capsules, while in other exemplary cases they support the transfer of the sperm into the receptaculum seminis (e.g., *Rhodnius prolixus*; Lococo and Huebner 1980) or adopt the function of a lubricant for the facilitated transport of the oocytes through an ovipositor (e.g., Orthoptera et al. 2000; Sturm 2008).

According to the results of previous studies (see review in Gillott 1988), the female accessory glands are commonly characterized by a simple structure, whereby their epithelium is composed of a cis-luminal cuticular intima, one or two cell layers, as well as a trans-luminal basallamina. If the gland epithelium includes only a single cell layer, the cells produce both the cuticula and the gland secretions. If, on the other hand, the gland epithelium consists of two cell layers, a division of labor may be observed, insofar as specific cuticulogenic cells are responsible for the formation of the intima and ductal structures running from the end apparatus of the secretory cells towards the gland's lumen. The secretory cells sensu stricto release their hydrophilic secretions into this ductal system (Sturm 2008).

Within the family of the Gryllidae, detailed investigations of the female accessory glands have thus far been largely restricted to Mediterranean and subtropical species such as *Teleogryllus commodus*, *Gryllus assimilis*, and *G. bimaculatus* (Sturm and Pohlhammer 2000; Sturm 2002b, 2008). For these species, gland morphology with single cell-layer epithelium was found, though significant discrepancies in gland shapes were detected. In the present study, the accessory glands in the female genital tract of the house cricket *Acheta domesticus*, a wide spread indigenous species, were subjected to a comprehensive study. In order to increase our knowledge of accessory glands, the respective organs of the female house cricket were investigated for their shape, size, morphology, and ultrastructure.

## Materials and Methods

### Animals

Crickets used for this study were reared in a climate chamber at the former Institute of Zoology, University of Salzburg. The following setup of environmental parameters was selected: environmental temperature at a constant 25 ^°^C, 12:12 L:D, 60 ± 10% RH. Adult animals were kept in lockable plastic boxes (50 × 30 × 20 cm) filled with a layer of peat soil measuring 3 cm in height. In order to provide a shelter to the crickets, empty egg cartons as well as wrinkled sheets of paper were placed into the boxes. The nutrition of the crickets consisted of fresh lettuce, standard diet for laboratory animals (Altromin®1222, www.altromin.de), oat flakes, and water offered via moistened cotton swabs.

### Preparation of the accessory glands

For the investigation of the accessory glands, females 5–10 days old were anaesthetized in a stream of CO_2_, decapitated, and transferred to a preparation tube filled with specific insect Ringer's solution (Sturm and Pohlhammer 2000). Subsequently, the abdomens of the specimens were opened on their ventral sides by conducting a cranio-caudal and two lateral sections. Fatty tissue and tracheoles were removed from the accessory glands. For the morphological and ultrastructural studies, they were removed from the crickets and put into interim storage using vessels filled with Ringer's solution.

### Fixation and microscopy of the accessory glands

For the preceding light-microscopic investigations, the accessory gland were transferred together with the Ringer's solution on a glass slide (75 × 25 mm) and studied under an interference-contrast microscope (Reichert Polyvar®, www.reichert.com). In order to optically enhance single components of the cells (e.g., nucleus, cuticula), the glands were additionally stained with the help of vital dyes such as carmine acetic acid. Photographic documentation of the glands took place using an analog camera and a related high-resolution black-and-white film (Agfa® APX-100, www.agfaphoto.com). The consistency of the secretion released from the insect organs was approximately determined by simple mixing experiments (e.g., with water or acetone).

Studies of the gland ultrastructure were mainly conducted with the help of the transmission electron microscope (TEM). Therefore, the dissected organs were fixed in a mixture of 2% paraformaldehyde and 2.5% glutaraldehyde (Karnovsky 1965). Both fixatives were buffered in 0.15 M sodium-cacodylate, thereby adjusting the pH exactly to 7.4. Fixatives and the buffer were subsequently calibrated to a concentration of about 325 mOsm using sucrose. After an immersion time of three hours, the fixed accessory glands were repeatedly washed in sodium-cacodylate buffer and post-fixed in 1% osmium-tetroxide for another two hours. The final stage of the fixation process included another washing procedure in the buffer as well as a dehydration of the fixed organs in a graded series of ethanol (70–100%). For pre-infiltration of the accessory glands, they were treated in graded mixtures of propylene-oxide and the medium used for their subsequent embedding (epoxy resin). Polymerization was realized by storing the specimens for 24 hours at 40 °C and for another 24 hours at 60 °C.

Semithin sections (thickness: 1–2 µm) of the studied organs were produced with a Reichert® OM-U2 microtome and subsequently mounted on a glass slide (75 × 25 mm) and stained with methylene-blue. Photographic documentation of the sections was conducted with the identical equipment as already used for the exploration of gland shapes. For the electron-microscopic work, ultrathin sections (thickness: ∼200 nm) were also produced with the Reichert® OM-U2 microtome and stained in uranyl acetate and lead citrate (Reynolds 1963). Final documentation was conducted with a Philips® EM-300 electron microscope (www.philips.com), using an accelerating voltage of 80 kV.

**Figure 1.**
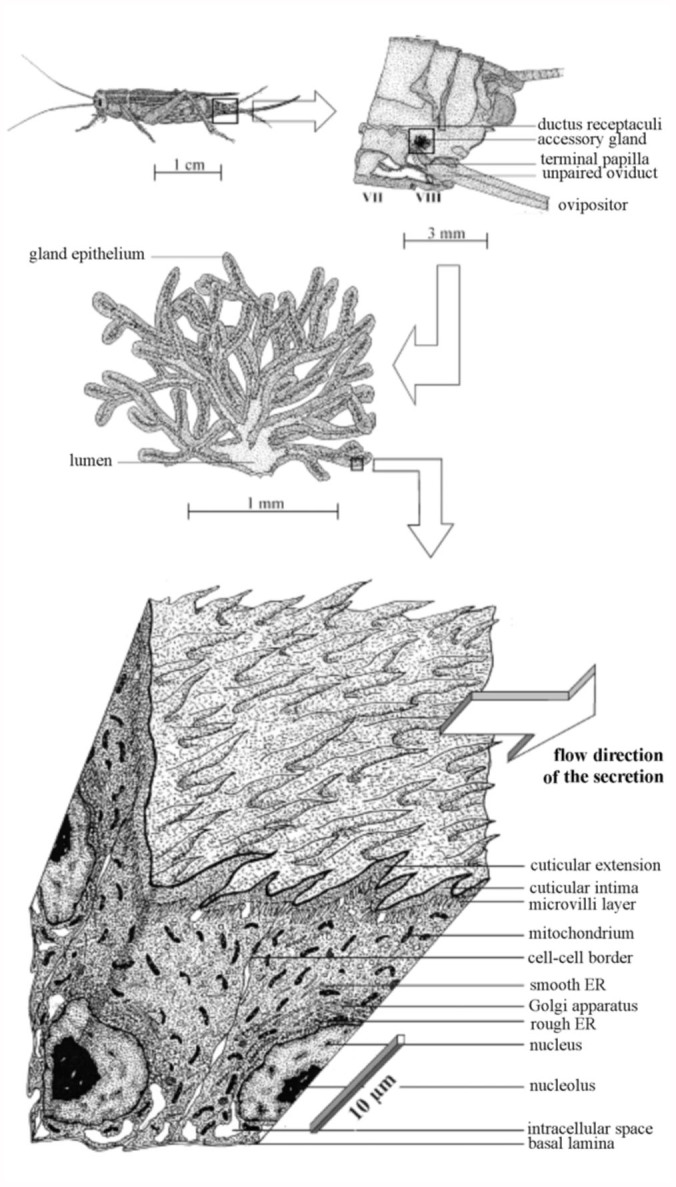
Position, shape, and cellular morphology of the accessory glands in female *Acheta domesticus*. High quality figures are available online.

## Results

### Position and shape of the accessory glands

**Figure 2.**
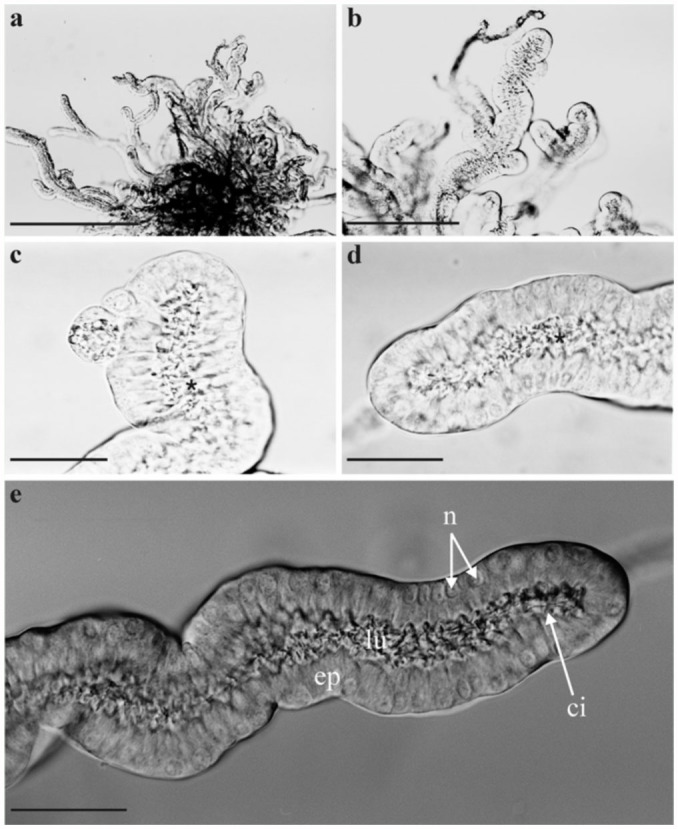
Morphology of the accessory glands of female *Acheta domesticus* with coarse subdivision into gland epithelium (ep) and lumen (lu): **(a)** overview (bar: 1 mm), **(b)** ramifications of the gland (bar: 0.5 mm), **(c)** and **(d)** terminal section of a single tubule with clear epithelium-lumen differentiation (bars: 0.2 mm), **(e)** interference-contrast image of a gland tubule showing additional morphological characteristics such as the basally situated nuclei (n) as well as the cuticular intima (ci) (bar: 0.2 mm). High quality figures are available online.

The female accessory glands of the house cricket commonly occur as pairs and are localized within the 7^th^ and 8^th^ abdominal segments (Figure 1). Their orifices are situated at that part of the genital chamber lying adjacent to the terminal papilla of the ductus receptaculi. The organs are usually covered by a dense network of tracheoles as well as some layers of fatty tissue. Their size, which can be determined under the microscope after their removal from the abdomen, varies with the age of the females and therefore ranges from 2 to 4 mm (Figure 1). The shape of the accessory glands is mainly characterized by numerous tubules (diameter: 0.2–0.3 mm), which form a complex system based upon dichotomous ramifications (Figures 1, 2a, 2b, 2c). The peripheral ends of the tubules are partly enlarged or again branch out into two separate terminal parts. The main branches of the organ merge into the basal region and release the produced secretion into the genital chamber via an orifice measuring 0.1–0.2 mm in diameter. The lumen of the gland is rather narrow in the single tubules (max: 0.1 mm), but enlarges significantly in the basal part of the organ.

### Morphology of the accessory glands

**Figure 3.**
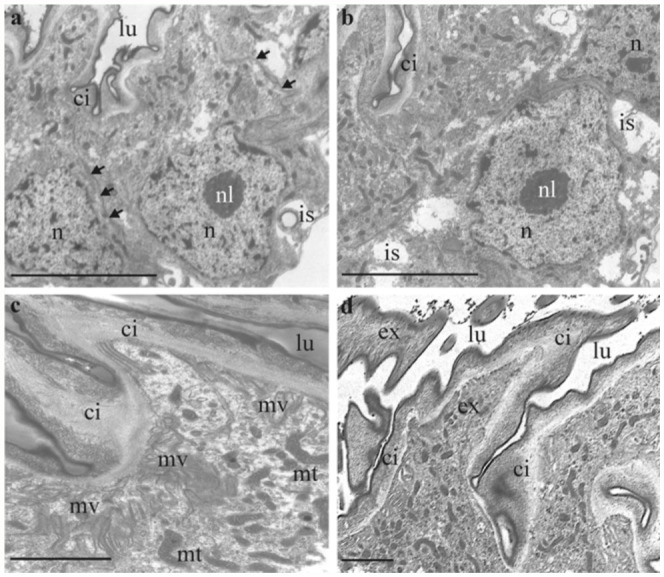
Transmission-electron micrographs showing the ultrastructure of single cells of the accessory glands of female *Acheta domesticus:* (**a**) and (**b**) gland cells with typical subdivision into a basal part with nucleus (n), intercellular spaces (is), and basallamina (bl) as well as an apical part with various cell compartments and microvilli layer; epithelial cells are demarcated from the lumen (lu) by a cuticular intima (ci); the arrows in (**a**) indicate cell-cell borders (bars: 10 µm); (**c**) detailed micrograph of the apical cell region including numerous mitochondria (mt), the sheet of microvilli (mv), microfilaments, vesicles, and lipid droplets (bar: 5 µm); (**d**) extensions formed by the cuticular intima (ex) (bar: 5 µm). High quality figures are available online.

Detailed studies under the light-microscope yield evidence that the female accessory glands of *A. domesticus* are constructed according to the simple schema introduced above which consists of a single layer of epithelial cells. The one-layer epithelium is accompanied by a cis-luminal cuticular intima as well as a trans-luminal basallamina (Figures 2d, 2e, 2f). The gland lumen itself serves in the collection of the secretion produced by the epithelium and its transport towards the orifice of the organ. The thickness of the cuticular intima varies between 0.5 and 3 µm. At specific sites, being in some parts a millimeter apart, it forms spiny or hair-like extension that deeply projects into the lumen and may reach a length of more than 10 µm (Figures 2e, 2f, 3c, 3d). The epithelial cell layer, being situated underneath the intima, includes columnar gland cells (Figure 2d), whose size (i.e., height × width) is characterized by remarkable uniformity throughout the gland. Accessory glands of young adults (pre-oviposition phase) differ from those of ovipositing females insofar as single epithelial cells of the organ measure about 20 × 10 µm in the first case and 35 × 20 µm in the second. The trans-luminal basallamina has a constant thickness of about 0.3 µm.

### Ultrastructure of the accessory gland cell

A key characteristic of the gland cells are their basally situated elliptic nuclei, making up about 20% of the cell volume. Besides a clearly developed nucleolus, they contain a rather high amount of genetically inactive hetero-chromatin. The nucleus is commonly surrounded by compartments necessary for both the synthesis of proteins (rough endoplasmic reticulum, Golgi apparatus) and the production of lipids (smooth endoplasmic reticulum). Additionally, the basal section of a gland cell includes local invaginations of the basal cell membrane that may form a wide system of intracellular spaces. The apical section of a gland cell is mainly composed of numerous mitochondria belonging to the cristae-type, protein-transferring vesicles, microfilaments, lipid droplets, and smooth endoplasmic reticulum. The apical cell membrane is re-structured to a dense layer of microvilli.

Based upon mixing experiments with water and acetone the secretion of the gland cells has to be evaluated as lipophilic, whereby its content of lipids is on the order of 65%. The mode, according to which the secretion passes the cuticular intima, has not been completely clarified thus far. However, due to the non-existence of breaks or channels within the cuticula, a purely diffusive transport at those sites (where the intima has its lowest thickness) is assumed.

## Discussion

In the study presented here the accessory glands in the female genital tract of *A. domesticus* were subjected to a detailed microscopic investigation. In the course of the study it was shown that the shape of the glands is commonly characterized by a complex system of tubules forming dichotomous ramifications. A similar complexity with regards to the external shape could be demonstrated for the accessory glands of *G. bimaculatus*, whereas the respective organs of *G. assimilis* and *T. commodus* have a much simpler structure (Sturm 2002a, 2002b, 2008; Sturm and Pohlhammer 2000). Concerning the external gland shape of *A. domesticus*, a certain analogy between females and males may be attested, insofar as the male accessory organs are also marked by an increased complexity of their external structure with respect to other orthopteran species (Hartmann 1970).

As exhibited in Figure 1, the size (height or width) of the accessory glands of the female house cricket varies between 2 and 4 mm-much smaller than the size of respective organs found in other cricket species. In the case of *G. bimaculatus*, for instance, fully differentiated, unwrapped glands may reach a height and width exceeding 10 mm (Sturm 2002). These inter-specific differences probably result from respective discrepancies in body size among the species. Besides a reduction of the gland size, the volumetric ratio between gland epithelium and lumen is significantly shifted in favor of the epithelium (Figure 2) in *A. domesticus*. As comprehensively discussed in the results section, the diameter of the lumen in single tubules takes a maximal value of 100 µm, but in most cases is smaller than the enclosing epithelium. A completely contrary situation may be found for the accessory glands of the cricket species that have been already noted above. Regarding *G. bimaculatus*, the lumina situated in the apical parts of single tubules may reach a width of 1 mm, thereby exceeding the height of the enclosing epithelium by a factor of 10–20 (Sturm 2002a).

From a morphological point of view the female accessory glands of *A. domesticus* are characterized by a rather simple scheme of construction including an inner cuticular intima, a single layer of epithelial cells, and an outer basallamina. This basic morphology, however, clearly corresponds with the respective construction scheme of other cricket species (Sturm 2002a) or insect groups (e.g., *Chironomus plumosus*; Wensler and Rempel 1962). A major specificity, which can be found in the accessory glands of all cricket species studied thus far, includes the hair-like or spiny cuticular extensions, which are partly characterized by projecting deeply into the lumen. The function of these extensions seems to be clearly recognizable in the case of the house cricket because besides a facilitation of secretory flow towards the orifice they mainly serve for the maintenance of a minimal luminal volume which guarantees the function of the glands if they are extremely wrinkled and intercalated between layers of fatty tissue. Similar extensions were also found in the receptaculum seminis of female *T. commodus* (Essler et al. 1992), where they probably support the movement of the sperm towards the ductus receptaculi.

The ultrastructure of single gland cells also shows a certain uniformity among the investigated cricket species, whereby each cell is commonly divided into two parts: (a) a basal part, containing the nucleus, regionally high amounts of intracellular spaces, and compartments for protein synthesis, and (b) an apical part, chiefly including compartments for protein and lipid synthesis, mitochondria, vesicles, microfilaments, and a layer of microvilli (Sturm and Pohlhammer 2000). The significance of the intracellular spaces probably lies in the enlargement of the basal cell surface, which allows an increased trans-luminal uptake of proto-substances being necessary for the production of the secretion. Similar processes of cell surface enlargement have been shown for several male accessory glands (Happ 1984), while respective observations in cells of female accessory glands are restricted to a few insect species (e.g., *Hyalophora cecropia*; Berry 1968).

The question regarding the passage of the secretion through the cuticular intima has not been clearly answered for *A. domesticus*, but it seems to work in a very similar way as in the case of the cricket species discussed above. Therefore, the lipophilic substance permeates the intima by simple diffusion (Sturm and Pohlhammer 2000). This kind of cuticular passage by lipophilic secretions seems to be widely distributed among insects, which is also shown by previous theoretical and experimental studies (Eidmann 1922; Koeniger et al. 1996). As demonstrated by numerous investigations, the release of hydrophilic substances from the producing gland cells, to the lumen always occurs by their transport through highly specific channel structures (e.g., Treherne 1957; Waku 1978; Essler et al. 1992).

The gland secretion is commonly thought to fulfill the following functions: first, it serves as a kind of lubricant for the facilitated transport of the oocytes through the ovipositor; second, it supports the introduction of the spermatophore tube into the ductus receptaculi; third, it supports the transport of the sperm into the receptaculum seminis. These main functions of the secretion have been also described in parts for non-endemic Mediterranean and subtropical cricket species (Sturm 2002a). Since the amount of secretion is significantly reduced in *A. domesticus* due to the limited gland size (see above), external functions of the secretion (e.g., protective substances for the oviposited eggs) may be largely excluded from these considerations. Based upon the comparison with other studies (e.g., Lococo and Huebner 1980), the highest plausibility is given for the secretion to act as a supporting substance during copulation.

## References

[bibr01] Berry SJ (1968). The fine structure of the colleterial glands of *Hyalophora cecropia* (Lepidoptera).. *Journal of Morphology*.

[bibr02] Brunet PCJ (1952). The formation of the ootheca by *Periplaneta americana* (L.). The structure and function of the left colleterial gland.. *Quarternal Journal of Microscopic Science*.

[bibr03] De Wilde J, Rockstein M (1964). Reproduction.. *The Physiology of Insecta*..

[bibr04] Eidmann H (1922). Die Durchlässigkeit des Chitins bei osmotischen Vorgängen.. *Biologisches Zentralblatt*.

[bibr05] Essler H, Herzog EM, Musiol IM, Pohlhammer K (1992). Morphology of the receptacular complex in the cricket *Teleogryllus commodus* (Saltatoria: Ensifera: Gryllidae).. *Entomologica Generalis*.

[bibr06] Gillott C, Adiyodi KG, Adiyodi RG (1988). Accessory sex glands in arthropoda - insecta.. *Reproductive Biology of Invertebrates III Accessory Sex Glands*.

[bibr07] Gillott C (1996). Male insect accessory glands: Functions and control of secretory activity.. *Invertebrate Reproduction and Development*.

[bibr08] Happ GM, King RC, Akai H (1984). Structure and development of male accessory glands in insects.. *Insect Ultrastructure*, *volume II*..

[bibr09] Hartmann R (1970). Experimentelle und histologische Untersuchungen der Spermatophorenbildung bei der Feldheuschrecke Gomphocerus rufus L. (Orthoptera, Acrididae).. *Zeitschrift für Morphologie der Tiere*.

[bibr10] Karnovsky MJ (1965). A formaldehyde-glutaraldehyde fixative of high osmolality for use in electron microscopy.. *Journal of Cell Biology*.

[bibr11] Kaulenas MS (1992). *Insect Accessory Reproductive Structures. Function*, *Structure*, *and Development*..

[bibr12] Koeniger G, Haenel H, Wissel M, Herth W (1996). Cornual gland of the honeybee drone (*Apis mellifera* L.): Structure and secretion.. *Apidologie*.

[bibr13] Leopold RA (1976). The role of male accessory glands in insect reproduction.. *Annual Reviews in Entomology*.

[bibr14] Lococo D, Huebner E (1980). The ultrastructure of the female accessory gland, the cement gland, in the insect *Rhodnius prolixus*.. *Tissue and Cell*.

[bibr15] Matsuda R (1976). *Morphology and Evolution of the Insect Abdomen*..

[bibr16] Reynolds ES (1963). The use of lead citrate at high pH as an electron-opaque stain in electron microscopy.. *Journal of Cell Biology*.

[bibr17] Sturm R (2002a). Morphology and ultrastructure of the female accessory sex glands in various crickets (Orthoptera, Saltatoria, Gryllidae).. *Deutsche entomologische Zeitschrift*.

[bibr18] Sturm R (2002b). Development of the accessory glands in the genital tract of female *Teleogryllus commodus* WALKER (Insecta, Orthoptera).. *Arthropod Structure and Development*.

[bibr19] Sturm R (2008). Morphology and histology of the ductus receptaculi and accessory glands in the reproductive tract of the female cricket, *Teleogryllus commodus*.. *Journal of Insect Science*.

[bibr20] Sturm R, Pohlhammer K (2000). Morphology and development of the female accessory sex glands in the cricket *Teleogryllus commodus* (Saltatoria: Ensifera: Gryllidae).. *Invertebrate Reproduction and Development*.

[bibr21] Treherne JE (1957). The diffusion of non-electrolytes through the isolated cuticule of *Schistocerca gregaria*.. *Journal of Insect Physiology*.

[bibr22] Tuzet O (1977). Les spermatophores des insectes.. *Traité Zoologie*.

[bibr23] Waku Y (1978). Fine structure and metamorphosis of the wax gland cells in a psyllid insect, *Anomoneura mori* Schwartz (Homoptera).. *Journal of Morphology*.

[bibr24] Wensler RJD, Rempel JG (1962). The morphology of the male and female reproductive system of the midge, *Chironomus plumosus* L.. *Canadian Journal of Zoology*.

